# Assessment of intensity effort of middle-aged adults practicing regular walking

**DOI:** 10.1590/bjpt-rbf.2014.0125

**Published:** 2015-10-09

**Authors:** Anderson A. Silva, Daniela A. Lima, Gabriella F. Vieira, Aline A. Fernandes, Danielle A. G. Pereira

**Affiliations:** 1 Departamento de Fisioterapia, Universidade Federal de Minas Gerais (UFMG), Belo Horizonte, MG, Brazil

**Keywords:** physical therapy, cardiovascular system, physical exertion, heart rate, walking

## Abstract

**Background:**

Walking is one of the most commonly recommended activities for sedentary individuals. When performed at the correct intensity, it can provide cardiovascular, respiratory, metabolic, and other benefits by providing a training effect in addition to reducing the risk of death from cardiovascular diseases and other chronic health conditions.

**Objectives:**

The primary aim of this study was to assess whether individuals who practiced regular unsupervised walking carry out the activity safely and with sufficient effort intensity parameters to have a positive physiological (training) effect. The secondary objective was to compare the training heart rate (HR) and the stability of the HR within the ideal range of training between the sexes.

**Method:**

Individuals were selected from walking tracks within the city of Belo Horizonte, Brazil. The study included subjects from 40 to 60 years of age who had practiced walking for at least two months prior to the study, walking at least three times a week. Individuals who agreed to participate in the survey were asked to walk 15 minutes at their usual pace with their HR measured every 5 minutes using a heart rate monitor. Their average walking HR was compared to the average training HR based on the formula: (220 - age) × 70 to 80% that would result in a positive physiological training effect.

**Results:**

Of the 142 individuals evaluated, 25.4% achieved the average training HR. This result was significantly lower than those who did not achieve the average training HR while walking (*p*=0.002). There were significant differences between men and women who had reached the training HR (*p*=0.0001).

**Conclusion:**

The authors found that individuals who walk regularly performed outside the range of the ideal HR intensity that would cause a positive physiological effect and therefore would probably not achieve a beneficial training effect while walking.

## Introduction

Cardiovascular diseases (CVD) are the leading cause of death in Brazil^1^, and its absolute risk increases progressively as the population ages^2^. Approximately one third of the acute myocardial infarcts (AMI) and a quarter of cerebral vascular accidents (i.e.stroke) occur in individuals under 65 years^1^. In addition, mortality from AMI and stroke is representative of the population between 35 and 64 years of age^1^. Around 11.6% of CVD deaths occur between 30 to 49 years and 35.7% between 50 and 69 years^1^. These data show that middle-aged individuals compose a population at risk for developing CVD, and consequently, early mortality.

The Unified Health System (Sistema Único de Saúde - SUS) features a number of sectorial policies that are designed to improve the health of individuals in Brazil. Among these policies, physical activity is presently one of the first priorities for action^3^. Therefore, a regular physical activity program is extremely important for maintaining health and is inversely associated with the risk of cardiovascular events^4^. To promote and maintain health, it is recommended that all healthy individuals between the ages of 18 and 65 years of age perform aerobic physical activity five days a week^5^. Studies have shown that physical activity of sufficient intensity to cause a positive physiological effect reduces the risk of developing CVD and other chronic diseases^5^^-^ 7, and reduces the risk of premature death from those diseases in healthy adults by about 50%^6^. Similar benefits have been found in individuals who already had the disease^6^. Physical inactivity is considered a relevant risk factor for the development of CVD, as are smoking and unbalanced diets^8^.

Research indicates that walking is one of the main physical activities recommended for sedentary individuals^9^. Leisure activity is the most commonly performed activity by adults and may therefore be an important component of physical activity programs^5^^,^ 10. It is a functional activity that provides health benefits while lowering the risk of injuries when compared to more vigorous activities^11^. It is also inexpensive and can be performed at any place or time, and is recommended for all age groups^11^^,^ 12.

The benefits of physical activity are influenced by the following factors: initial level of aerobic fitness, and, frequency, duration and intensity of training^7^. Among these, the intensity of training is an important factor for promoting positive adaptations from the exercise^7^^,^ 11.

There is evidence in the literature that the intensity of physical activity is inversely and linearly associated with mortality^6^. Adults between the ages of 18 and 65 years should perform aerobic exercise of moderate intensity for at least 30 minutes, five times a week or vigorous aerobic exercise for 20 minutes, three times a week^5^. The performance of moderate intensity activity has been linked to a reduced risk of AMI in healthy adults^5^ and to a lower risk of developing Type II Diabetes in middle-aged men^6^. Hamer and Chida^13^ found a lower risk of CVD and mortality for both men and women who walked regularly. Significant effects were observed in the rate of walking compared to the volume of the exercise^13^. This information suggests that exercise intensity is a more sensitive predictor of positive clinical outcomes.

There are several ways to calculate the intensity of the physical activities, such as: energy expended over time, absolute level of exercise or power production, percentage of maximum oxygen consumption (VO_2max_), exercise in relation to the lactate threshold, heart rate (HR) reserve or percentage of maximum heart rate (HR_max_), multiple metabolic rate, and perceived effort^7^. Many studies have used the percentage of VO_2max_. However, to use this measurement, it is necessary to perform an exercise test using expired gas analysis, which makes this method less accessable to the general population^7^. Despite this limitation and due to the linear relationship between exercise intensity and HR, the intensity can be measured in HR values equivalent to VO_2max_ 11.

Since physical activity is encouraged by the health promotion policies of the SUS^3^ and its benefits have been extensively proven^4^^-^ 6 ^,^ 13, it is important to know whether individuals who do physical activity are performing at the proper intensity to achieve major cardiovascular gains. In addition, it is also important to evaluate how the strategy of engaging in physical activity for health promotion as proposed by SUS has been effectively achieved in Belo Horizonte, Brazil as well as in other parts of the country. Thus, the primary objective of this study was to evaluate whether individuals who walk regularly as an unsupervised activity are carrying out this activity within the parameters of safety and with sufficient effort intensity (training HR: defined as 70-85% of maximum HR_max_) to achieve a positive physiological (training) effect. The secondary objective was to compare, between the sexes, the training HR and its stability in the positive physiological effect range.

## Method

This is a descriptive observational study approved by the Research Ethics Committee of Universidade Federal de Minas Gerais (UFMG), Belo Horizonte, MG, Brazil (CAAE 0501.0.203.000-11).

The sample size was calculated according to a formula and was based on the estimated proportion of the population (n=Z^2^_α/2_ .p.q/ E^2)^; where n is the number of individuals in the sample; Z_α/2_ is the critical value corresponding to the level of confidence desired; p is the proportion of individuals within the age group studied that walked regularly in Belo Horizonte; q is the proportion of individuals who walked regularly but were outside the age group in question (q=1 - p) and E is the estimated margin of error. The values of p and q were unknown, therefore both were set to 0.5. The α value was set to 0.05, thus Z_α/2_=1.96. The value of E chosen was 10% (0.1). Replacing these values in the formula, a sample size of 97 individuals was determined to be necessary to show the significance of any results.

Subjects were recruited from walking tracks from each of the nine regional districts of Belo Horizonte, MG, Brazil. Data collection occurred in the early morning or late afternoon and early evenings, from Monday to Friday. The goal was to achieve the estimated sample size and represent each of the nine districts. A total of 146 individuals were evaluated.

The inclusion criteria were: individuals between 40 to 60 years of age, apparently healthy, and who walked regularly for at least two months prior to the study with a frequency of at least three times a week. Subjects were excluded from the study if they had a diagnosis of heart failure, peripheral arterial disease, coronary arterial disease or chronic obstructive pulmonary disease (COPD), were taking beta-blockers and/or had a pacemaker, had a systolic blood pressure (BP) above 140 mmHg, and/or diastolic above 100 mmHg. A comparison of the walking effort was conducted between the time of the test and the subject's usual walking level intensity to exclude individuals who, in the presence of a healthcare professional, walked at a slower pace than usual or, instead, walked at a faster pace than usual. The goal was to select individuals who performed the test within the usual parameters of their regular walking activity. Thus, if, at the time of completion of the test, the subjects reported being "much less tired" or "much more tired" compared to their usual intensity of walking, they were also excluded from the study. For the selection of subjects and subsequent data collection, a semi-structured questionnaire was employed, in which the subjects were identified only by their age and sex, and additional data collected included: HR_max_; training HR; presence of the medical conditions noted above and/or whether the subject had a pacemaker; had hypertension; used medications; when they started walking and weekly frequency; how they controlled their walking practice; reasons for walking; any previous orientation on how to exercise with the correct HR intensity; whether they practiced any other physical activities; comparison of the walking intensity during the data collection day compared to their other days of walking, and, measurement of blood pressure (BP).

The participants were invited, *in loco*, to respond to the questionnaire. At this point, if they wanted to participate and met the inclusion criteria, they were asked to sign a consent form. They were then asked to walk for 15 minutes, at their normal pace on a flat track surface. Their HR was measured every 5 minutes and the average of the three measurements was used for the analysis. For the HR measurement, a Polar FT1 heart rate monitor with a coded chest belt (Polar T31) was used, and a stethoscope and Littmann sphygmomanometer were used to measure blood pressure.

The average HR obtained from the subjects was compared with the average of the training HR needed to produce a positive physiological effect during walking - 70-85% of maximum heart rate - in order to determine whether an intensity that caused a positive physiological effect was reached while walking.

Data were presented as mean and standard deviation for continuous variables and as frequency for categorical variables. For the comparative analysis between the percentage of subjects who walked or did not walk within the training HR, a chi-square test was used. An independent t-test was used to compare age and average heart rate during the activity. The analysis was conducted using the total sample, stratified by region and gender. The level of confidence was set at 5%. For all analyses, the Statistical Package for Social Sciences (SPSS) version 15.0 was used.

## Results

A total of 146 volunteers participated in the interview. Of these, four participants were excluded because they claimed to be much less tired after the 15-minute walk test. Thus, 142 people met all the criteria and participated in the study.

For the nine districts of the city of Belo Horizonte, 16 subjects came from each of the northeast, north, northwest and Barreiro districts, 15 subjects came from each of the eastern, west, Venda Nova and Pampulha districts, and 18 subjects came from the south-central region.

The average age of the individuals was 51.01±5.49 years, 46.5% were male and 53.5% female and no significant difference in age between the sexes was found (*p*=0.136). Table 1shows the comparison between sexes of the variables age and the average training HR reached during walking. Of the total, 20.4% were controlled hypertensive and 47.9% of respondents were using some kind of medication. The average systolic BP found was 120.32±10.81 mmHg and the diastolic BP was 81.38%±9.23 mmHg. 

**Table 1.** Comparison of age, heart rate and whether a training heart rate was reached between the sexes for people using walking as an exercise.

	**Men (n=76)**	**Women (n=66)**	***p*-value**
**Age (years)**	51.64±5.35	50.29±5.60	0.143*
**HR (bpm)**	113.82±12.73	105.33±11.71	0.0001*
**Reached a sufficient HR to cause a training effect**			0.0001**
**Yes**	29	7	
**No**	47	59	


*Independent t-test; **chi-square test (p<0.05). HR: heart rate; bpm: beats per minute.

Regarding the time subject walked, 78.2% had been walking for more than six months, 0.7% for six months, 1.4% for five months, 2.8% for four months, 6.3% for three months, and 10.6% for two months. For weekly walking frequency, 88% walked three, four or five times a week and the remaining 12% walked six or seven times per week.

With respect to how the participants controlled the amount of their walking activity - participants could choose more than one option - 66.9% used time as the factor for how long they walked, 43.7% used distance for how long they walked, and 4.9% had no specific way of controlling how long they walked in a session. None of the participants selected the option of using a device to measure the level of intensity of their walking (e.g. heart rate monitor).

When asked what motivated the subjects to walk as an exercise - 85.2% reported they were walking for health reasons, 58.5% for esthetic reasons, 66.9% for fitness, 81.0% for pleasure, and 6.3% for other reasons (subjects could pick more than 1 option). Additionally, 26.1% had been taught how to determine the correct intensity while walking and 33.1% also practiced other physical activities.

It was found that 25.4% of the subjects reached the training HR which had a positive physiological effect. The number of participants who walked at a rate that did not have a positive physiological effect was significantly higher than the number of subjects who walked at a pace that had a positive physiological effect (Table 2). About 10% of the women and 38% of men walked at a pace that had a positive physiological effect. When these two groups were compared, a significant difference was observed (Table 1). 

There were no significant differences between the districts of the city in relation to the percentage of those who walked at a pace that had a training effect and those who did not (*p*=0.655). Table 2 shows the results in percentage by each city district. Of the 37 subjects who received orientation regarding how to determine if they were walking at a pace that would have a positive physiological effect, 11 reached the desired pace, with no significant difference between this group and those who had not been through an orientation about walking at a HR that had a training effect (*p*=0.513). 

**Table 2.** Relative and absolute frequency of the individuals who reached a sufficient HR to cause a training effect, stratified by regional municipality.

**District**	**Reached a training effect HR**	**Absolute frequency (number)**	**Relative frequency (%)**
Northeast	Yes	4	25
	No	12	75
East	Yes	3	20
	No	12	80
Barreiro	Yes	6	37.5
	No	10	62.5
Central-South	Yes	7	38.9
	No	11	61.1
Venda Nova	Yes	2	13.3
	No	13	86.7
North	Yes	2	12.5
	No	14	87.5
West	Yes	4	26.7
	No	11	73.3
Pampulha	Yes	5	33.3
	No	10	66.7
Northwest	Yes	3	18.8
	No	13	81.3
Total	Yes	36	25.4*
	No	106	74.6


HR: heart rate. *statistically significant difference (*p*=0.002) by the chi-square test.

## Discussion

The goal of this study was to verify whether middle-aged individuals who practiced walking as a regular activity in Belo Horizonte walked at the intensity determined as being of sufficient intensity to cause a positive physiological effect - 70-85% of maximum heart rate - and if there was a difference between the sexes regarding exercising at an intensity that would cause a positive physiological effect. From the results, it was possible to characterize the sample studied and determine that most subjects didn't work at sufficient intensity that would cause appositive physiological effect. It was also observed that men and women behaved significantly different during this exercise. Higher percentage of men reached target HR during exercise.

Individuals who used beta-blockers were excluded, since these drugs reduce the HR, inotropism (affect muscle contractility) and BP. Thus, individuals who use beta blockers have more difficulty reaching the target HR during exercise^11^^,^ 14 ^,^ 15. According to the VI Brazilian Guidelines on Hypertension, individuals are not advised to exercise when the systolic and diastolic BP are above 160 and/or 105 mmHg, respectively. In this study, in order to increase the safety of the participants, BP values below 140/100 mmHg were also an exclusion criterion.

The sample was composed mainly of females, apparently healthy, who had practiced walking regularly for more than six months, three to five times a week. The length of the activity was measured by time and was executed by the subjects for health purposes and enjoyment. Most of the subjects had never received orientation on the recommended intensity for walking and did not engage in other physical activity. Similar characteristics have been reported in a study conducted using 430-interviewed subjects^16^. The authors also found that most participants were female, apparently healthy, who performed physical activity five times a week for over a year and were walking for health reasons and to get fit, without professional guidance and did not practice other physical activity^16^.

The literature is extensive on HR ranges that result in beneficial effects of the cardiovascular system^4^^-^ 6 ^,^ 13. The range of physical activity considered moderate intensity varies considerably within the literature^5^^,^ 7 ^,^ 11 ^,^ 13 ^-^ 15 but spans from 60-85% of the HR_max_. Faced with the need to define a specific HR range for this study, the authors defined the training heart rate that had a positive physiological effect as a sensitive zone of training in the range from 70 to 85% of HR_max_, which corresponds to 60 to 80% of VO_2max_ and 60 to 80% of the HR reserve^7^^,^ 11. Thus, the range of 70 to 85% of HR_max_ was determined, in this study, as the training HR, or the training range in which the subjects would be walking at an intensity that had a positive physiological effect.

The results also showed that most of the subjects evaluated did not reach a HR that had a positive training effect. This means that these subjects were not getting the benefits of an exercise performed at an ideal intensity^6^^,^ 7 ^,^ 14.

Initially, the absence of professional guidance and monitoring could have been one of the contributing factors to the greatest number of subjects who walked with insufficient intensity to cause a positive physiological effect. From the questionnaire employed, it was impossible to detect the actual level of knowledge of the subjects regarding the appropriate intensity to have a positive physiological effect, but the literature shows the influence of professional monitoring on the effectiveness of exercise^17^. A study conducted on 20 men and 25 women, with the goal of investigating the relationship between an individualized exercise prescription and an efficient walking program, showed that without professional orientation, 70% of the subjects did not reach an appropriate exercise intensity^18^. After the participants received professional guidance and exercised under an appropriate intensity, an increase in the respiratory capacity of all participants was observed^18^. Therefore, it was concluded that professional orientation was important to enable the participants to obtain the maximum health benefits of physical activity.

Some studies stated that to obtain the benefits of exercise, it is recommended that individuals perform moderate activity for at least for 30 minutes, such as walking, five or more days a week^9^^,^ 15 ^,^ 17. However, most of the population failed to follow this recommendation or did not understand what "moderate activity" meant in terms of intensity^17^. One recommendation would be for public agencies to hire trained professionals to monitor and instruct the population regarding exercise intensity, use of properly prescribed exercises and to educate the individuals about their limits, signs and symptoms related to the activity and the proper exercise effort. To prescribe or practice exercise, the principle of intensity should be considered^19^.

Individuals should know about correct exercise intensity if they want to accomplish a training or positive physiological effect. This may be accomplished by performing an ergometric stress test to determine the HR peak^15^. However, in the absence of a stress test, the formula use in this study can be easily applied. Other ways to guide individuals in achieving a training effect would be to do an activity at an intensity that just allows the person to speak full sentences without interruptions^15^or to perform moderate aerobic activity ranging from 12 to 16 on the Borg Scale of Perceived Exertion (between relatively easy and very tiring)^19^.

Another factor that could impose difficulties for individuals to achieve a training effect is that for many walking tracks on city streets, participants need to cross intersections, which can slow down their pace of walking. This was verified in our study, as the districts with the highest percentage of subjects practicing walking at the desired training HR were those in which the participants did not have to stop the activity to cross a public road. Some studies have addressed the need to create tracks for walking, but unfortunately information on how car traffic could influences the pace of walking activity is still lacking^8^^,^ 9 ^,^ 20. Places with more sidewalks, with forested areas, and with less vehicle traffic, are more pleasant and safe to walk and could positively influence the physical activity levels of the population^8^.

Another important point of this paper is the statistically significant difference found between men and women in walking at a pace that had a training effect. One hypothesis that could explain this finding is that males tend to perform more vigorous activity (such as running) than females. In a study that questioned participants on the intensity of exercise (i.e. walking, trotting or running), the majority of those who were trotting or running were men^16^. In addition, among men, the activities were predominantly walking, soccer, running, tennis, volleyball, wrestling and weight training. Among women, walking, gymnastics, dance and aerobics were more common^21^. Thus, it could be inferred that the activities performed by men are more vigorous than the ones performed by women.

Other factors may have influenced the results of this study, such as the lack of proper knowledge on how to achieve an ideal training HR during exercise and while walking on city streets and having to cross streets, requiring individuals to slow down or even stop for a traffic light in order to cross the road. However, in this study, these factors were not analyzed but the results could easily be generalized to the Brazilian urban population.

In face of the SUS's strategy of promoting a habit of physical activity in the Brazilian population, the results of this study are valuable since they show the importance of using health professionals to review exercise guidelines and to educate the population on how to achieve an optimal positive physiological effect during walking. It is important to help walkers achieve greater cardiovascular benefits using parameters such as a target training HR or percentage of VO_2max_. In addition, studies that address the hypotheses raised in this study are fundamental. The results of this research could be used to develop strategies to instruct individuals and to inform public policies on the importance of walk at a sufficient intensity to have a beneficial effect on cardiovascular responses.

## Conclusion

Most middle-aged individuals, who practiced walking regularly, do so at an intensity that was not sufficient enough to obtain health benefits from the activity. It was also observed that men are more likely to work at the appropriate HR to have a positive physiological effect than women.
